# Nicotinamide (Vitamin B3) Deficiency in Follicular Fluid of Patients With Ovarian Ageing

**DOI:** 10.1111/jcmm.71085

**Published:** 2026-03-19

**Authors:** Cinzia Bocca, Magalie Boguenet, Lisa Boucret, Pierre‐Emmanuel Bouet, Pascal Reynier, Pascale May‐Panloup

**Affiliations:** ^1^ Mitolab, MitoVasc Institute, CNRS 6015, Inserm U1083 Angers University Angers France; ^2^ Biochemistry and Molecular Biology Laboratory Angers University Hospital Angers France; ^3^ Reproductive Biology Laboratory Angers University Hospital Angers France; ^4^ Department of Gynecology and Obstetrics Angers University Hospital Angers France

**Keywords:** 1‐methylnicotinamide, follicular fluid, mitochondria, nicotinamide, ovarian ageing, vitamin B3

## Abstract

Nicotinamide is an essential precursor of NAD involved in energy metabolism. A decline in NAD levels across tissues during ageing is well‐documented, and NAD metabolism has been implicated in ovarian ageing. We investigated the relationship between ovarian ageing and the levels of nicotinamide (NAM) and its metabolite 1‐methylnicotinamide (MNA) in follicular fluid (FF) and blood of IVF patients. FF was obtained from 86 patients with ovarian ageing and 82 controls. Serum samples were collected from 24 and 29 patients, respectively. Nicotinamide and 1‐methylnicotinamide levels were quantified by HPLC‐MS/MS. Nicotinamide concentration was significantly decrease in the FF of patients with ovarian ageing (*p* < 0.01), associated by an increase in 1‐methylnicotinamide concentration (*p* = 0.01), leading to a sharp increase of the MNA/NAM ratio (*p* < 0.001). This ratio negatively correlated with antral follicle count, anti‐Mullerian hormone level and the number of oocytes retrieved, while positively correlating with age. Additionally, there was a positive correlation between the ratio values in FF and serum. Our results suggest an imbalance towards the nicotinamide degradation pathway in ovarian ageing, potentially impairing the NAD salvage pathway. They argue in favour of using nicotinamide to counteract ovarian ageing in patients and suggest the assessment of MNA/NAM ratio as a biomarker.

## 1 Introduction

Female fertility exhibits a gradual decline commencing around the age of 30, with a pronounced acceleration observed between 35 and 37 years of age [1]. This decline is associated with an increased risk of miscarriage and a higher prevalence of congenital anomalies [2]. Concurrently, the average maternal age continues to rise in contemporary societies, positioning age‐related infertility as an escalating public health issue. Importantly, the effectiveness of assisted reproductive technologies (ART) remains limited in the context of ovarian ageing, highlighting the urgent need for enhanced mechanistic insights and the identification of reliable biomarkers for this condition.

Ovarian ageing is characterised by a decline in oocyte quality, which exerts a direct influence on embryonic developmental potential. Mitochondria are central to oocyte competence [3], primarily through their role in ATP generation via oxidative phosphorylation. This bioenergetic process relies on the redox cofactors NAD^+^/NADH_2_ (oxidised and reduced forms of nicotinamide adenine dinucleotide) and FAD/FADH_2_ (flavin adenine dinucleotide) which transfer electrons from energetic reduced substrates to the mitochondrial respiratory chain allowing oxidative phosphorylation and ATP production. NAD is also important for genomic integrity and the regulation of epigenetic homeostasis [4]. Mitochondrial dysfunction and NAD depletion are well‐established hallmarks of ageing across various tissues, including the ovary [5, 6]. In murine models, reduced ovarian NAD^+^ levels have been correlated with compromised oocyte quality [7, 8], and genetic disruption of NAD^+^ biosynthetic pathways has been shown to accelerate ovarian ageing [9].

Despite its biological relevance, direct quantification of NAD in human ovarian tissue presents significant challenges. NAD is an intracellular molecule with a short half‐life and is typically released into biological fluids only under conditions of cell necrosis [10]. Consequently, its measurement in follicular fluid (FF) is not reliable. Moreover, ethical and technical constraints preclude direct NAD quantification in human oocytes, particularly given the limited sensitivity of current detection methods, which would require pooling multiple oocytes [11].

To circumvent these limitations, attention has turned to nicotinamide, a precursor of NAD, and its methylated metabolite, 1‐methylnicotinamide. Nicotinamide (niacinamide) is one of the two components of vitamin B3 with niacin and has been positively correlated with oocyte maturity and fertilisation outcomes in IVF patients [12]. Metabolomic analyses have further revealed dysregulation of nicotinamide metabolism in women with poor ovarian response, a phenotype commonly associated with ovarian ageing [13]. Although previously considered biologically inert, 1‐methylnicotinamide has since been attributed anti‐inflammatory and vasoprotective properties [14, 15].

The present study aims to investigate the relationship between ovarian ageing and the concentrations of nicotinamide and 1‐methylnicotinamide in the FF of women undergoing IVF. Secondary objectives include evaluating the association between nicotinamide levels and clinical parameters, and exploring the correlation between nicotinamide concentrations in FF and peripheral blood.

Ovarian ageing is characterised by both qualitative and quantitative changes in ovarian reserve but its characterisation remains complex and subject to debate. Some authors rely solely on chronological age, using threshold values that are still not universally agreed upon [16, 17]. Other ones emphasise the significant interindividual variability in ovarian ageing, highlighting the influence of genetic and environmental factors. They argue that relying solely on chronological age is insufficient, and that additional biomarkers are needed for a more accurate assessment [18, 19, 20]. To account for this inter‐individual variability, we stratified patients based on their ovarian status into two groups: those with a normal ovarian profile (control group: CTRL) and those exhibiting signs of ovarian ageing (OA), as defined by established clinical and biological criteria.

## 2 Materials and Methods

### 2.1 Study Design and Population

**FIGURE 1:**
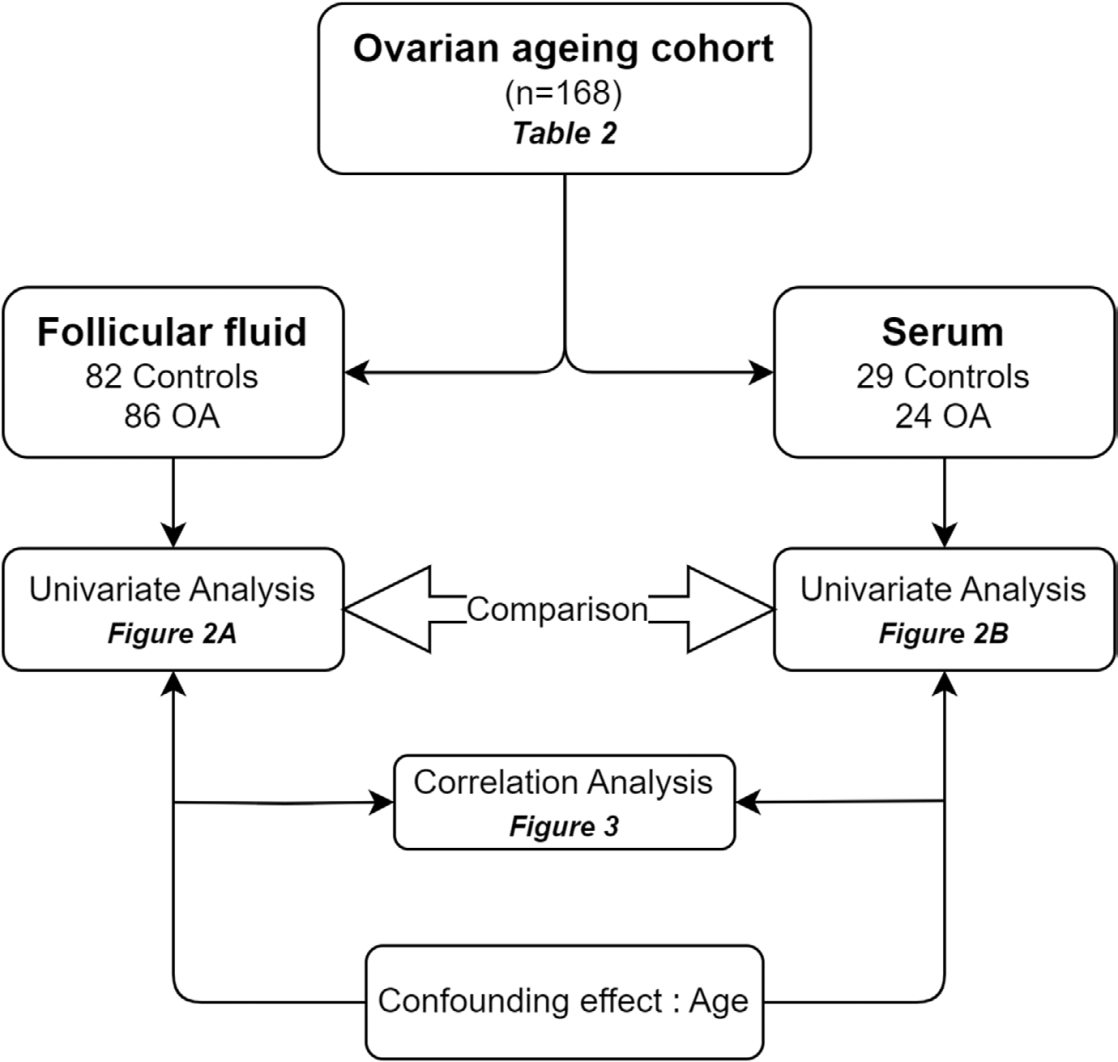
Decision flowchart. Each step of the workflow described in the paper is annotated with the corresponding figure or table number when applicable. OA, ovarian ageing profile.

This retrospective cohort study was conducted at the ART centre of the University Hospital of Angers, France, between March 2016 and December 2021. A total of 168 IVF cycles were included, with FF obtained from 86 patients with ovarian ageing (OA) and 82 control patients. Additionally, serum samples were collected from 53 of these patients, including 24 patients with OA and 29 control patients, between June 2020 and December 2021 (Figure 1).

The ovarian profile was assessed based on age, baseline follicle‐stimulating hormone (FSH) levels, antral follicle count (AFC) and anti‐Mullerian hormone (AMH) levels. According to the Bologna criteria [21] and POSEIDON classification [22], OA was defined by the presence of at least two of the following criteria: advanced maternal age (≥ 40 years), ≤ 3 oocytes retrieved in the current or previous cycles, and abnormal ovarian reserve test results (i.e., AMH < 1.2 ng/mL and/or AFC < 7 follicles and/or FSH ≥ 10).

To be classified in the control group, women were required to meet no more than one of the criteria mentioned above, and they were undergoing treatment for isolated male infertility (OAT or azoospermia).

Exclusion criteria were known risk factors for genetic, surgical or iatrogenic ovarian insufficiency. Additional exclusion criteria were as follows: (1) Polycystic ovary syndrome (PCOS); (2) Endometriosis; (3) Egg donation; (4) Age below 18 or over 43 years (the age limits for access to IVF treatment in France); (5) Patient's refusal to consent to the processing of their personal data.

### 2.2 Controlled Ovarian Stimulation

The IVF stimulation protocols were selected by primary physicians based on factors such as AFC, AMH, age, body mass index (BMI) and the ovarian response observed in previous IVF cycle(s). Most cycles (91%) used GnRH antagonists following estrogen priming. In an alternative approach, pituitary suppression was achieved using a gonadotropin‐releasing hormone agonist. Ovarian stimulation was accomplished through the administration of recombinant FSH and/or human menopausal gonadotropin, with doses ranging from 125 to 450 IU per day. Ovarian response was monitored through transvaginal ultrasonography and serum estradiol measurements.

### 2.3 Collection of Follicular Fluid

Ovulation triggering was conducted using 6500 IU of recombinant human chorionic gonadotropin (rhCG) and/or 0.2 mg of triptorelin when at least three follicles reached a diameter of 17 mm. Oocytes were retrieved transvaginally under ultrasound guidance 36 h later. The retrieval procedure took place in the morning under general anaesthesia, requiring patients to fast prior to the procedure. Following retrieval, the oocytes were isolated from the FF for fertilisation (IVF or ICSI) and subsequent culture. The remaining FF from each patient was pooled and immediately centrifuged at 600 g for 10 min to eliminate erythrocytes and leukocytes. The supernatant was collected and stored frozen at −80°C until further analysis.

### 2.4 Collection of Serum

Blood samples were collected on the day of oocyte retrieval. The serum was stored at −80°C until use.

### 2.5 Ethical Approval

All participants had signed the informed consent prior to inclusion, and the collection of samples was approved by the Committee for the Protection of Individuals of Angers University Hospital, France (Number DC‐2014‐2224). This study has also been approved and registered by the institution's ethics committee under reference number 2025‐221.

### 2.6 Nicotinamide and 1‐Methylnicotinamide Quantification

30 μL of serum or follicular fluid, fortified with 10 μL of the isotope metabolite standards in methanolic mixture (nicotinamide‐2,4,5,6‐d4 and N‐methyl‐13C, d3‐nicotinamide at 10 μg/mL, 98% purity, Sigma Aldrich, St. Quentin Fallavier, France), were extracted with a methanol/water (95/5) solution (Optima LC/MS grade, Fisher Scientific, Illkirch, France). Following vortexing and centrifugation, supernatants were evaporated to dryness at room temperature, and reconstituted samples (300 μL acetonitrile/water 90/10, Optima LC/MS grade, Fisher Scientific, Illkirch, France) were injected into an Agilent 1200 HPLC system coupled with a Sciex Qtrap 5500. Chromatography elution was performed on an HILIC Kinetex column (1.7 μm, 50 mm × 2.10 mm, 100 Å, Phenomenex, Le Pecq, France) maintained at 40°C. A ramp gradient was employed, starting with mobile phase A (acetonitrile/water, 90/10 with 0.2% formic acid and 100 mM of ammonium formate, Optima LC/MS grade, Fisher Scientific, Illkirch, France) and ending with 70% of aqueous phase B (with 0.1% of formic acid and 100 mM of ammonium formate), with a flow rate of 0.4 mL/min over a total runtime of 12 min. The HPLC autosampler temperature was set at 4°C. Multiple reaction monitoring (MRM) detection in positive ionisation mode was employed, utilising the transitions of interest and collision energies as summarised in Table 1.

**TABLE 1:** Mass and voltage per compound.

ID	Q1 mass (Da)	Q3 mass (Da)	CE (volts)
Nicotinamide	123.055	80.05	30
1‐methylnicotinamide	137.071	78.034	35
Nicotinamide d4	127.080	84.075	25
N‐Methyl‐^13^Cd_3_‐nicotinamide	141.090	110.060	25

Abbreviation: CE, collision energy.

Injections sequences were carried out as follows: after calibration points and four quality level controls (QC) prepared on mobile phase (1‐methylnicotinamide chloride and nicotinamide from Sigma Aldrich, St. Quentin Fallavier, France, 98% purity), biological samples were injected sequentially, with a maximum of 25 samples processed before performing a quality control reinjection. The linearity of nicotinamide was confirmed within the range of 2–40 ng/mL, with a lower limit of quantification (LLOQ) set at 0.5 ng/mL and a calculated limit of detection (LOD) of 0.32 ng/mL. Similarly, the linearity of 1‐methylnicotinamide was validated between 5 and 100 ng/mL, with a LLOQ of 2.5 ng/mL and an LOD determined to be 0.17 ng/mL.

### 2.7 Statistical Analysis

Quantitative variables of patients were analysed using the non‐parametric Mann–Whitney test, while qualitative variables were assessed using the chi‐square or Fisher's exact test. These analyses were performed using GraphPad Prism, version 8.0 (GraphPad Software, San Diego, CA, USA), with significance set at a *p‐*value < 0.05 for all tests.

MetaboAnalyst 6.0 was used for statistical analyses on raw data. Univariate analyses were conducted using the non‐parametric Wilcoxon rank‐sum test, with fold changes (FC) reported as the ratio between the ovarian ageing and control subjects. The Spearman rank correlation coefficient (Pattern Search module, MetaboAnalyst) was used to explore potential associations between the two molecules, their ratio and clinical data. Only metabolite‐clinical associations with an absolute correlation coefficient greater than 0.3 and a significant FDR‐adjusted *p*‐value (Benjamini‐Hochberg correction) were retained. The relationship between the MNA/NAM ratio calculated in follicular fluid and the ratio determined in the serum of the same patients (*n* = 53) was evaluated using Spearman's rank correlation to assess the strength and direction of the association.

To assess the respective effects of age and ovarian profile on the MNA/NAM ratio, we performed complementary analyses using GraphPad Prism, version 8.0. A linear regression model was developed to evaluate the association between the MNA/NAM ratio, ovarian function and age. Collinearity was assessed using Variance Inflation Factor (VIF) diagnostics, with VIF values greater than 5 indicating substantial multicollinearity.

To control for age‐related confounding effect, propensity score matching was employed using the MatchIt package (version 4.5.0) in RStudio 2023.06.0 Build 421 (R version 4.3.0). Participants were matched 1:1 based on age using the nearest‐neighbour method with a calliper width of 0.2 (standard deviation of the logit of the propensity score). Balance diagnostics, including standardised mean differences (SMD; SMD < 0.25 as adequate balance) and variance ratios, were assessed before and after matching to ensure comparability between the OA and N groups. Finally, the non‐parametric Mann–Whitney test was used to compare the MNA/NAM ratios between the OA and N groups in the matched sample (*p‐*value < 0.05), using GraphPad Prism, version 8.0.

## 3 Results

### 3.1 Patients' Characteristics

In accordance with the inclusion criteria, AMH and AFC levels were significantly lower in the OA group compared to the N group, while the FSH levels and age were significantly higher. These parameters were specifically used to characterise our two populations. Unsurprisingly, the doses of FSH administered during the stimulation phase were higher in the OA group. However, there were no significant differences observed in the drugs used to suppress LH between the two populations. Similarly, there were no significant differences in terms of BMI or tobacco smoking addiction, both of which can potentially impact ovarian reserve. The number of oocytes retrieved was significantly lower in the OA group, reflecting the reduced ovarian reserve in this group (Table 2).

**TABLE 2:** Characteristics of patients.

		Control group *n* = 82	OA group *n* = 86	*p*
Age (years)		32.3 ± 3.8	36.6 ± 4	< 0.0001
BMI (kg/m^2^)		24.6 ± 5.3	24 ± 4.8	0.46
Smoking status	Nonsmoker	72 (88)	76 (88)	0.53
	Smoker	7 (9)	9 (11)	
	Information missing	3 (3)	1 (1)	
Baseline FSH (IU/L)		6.9 ± 2	9.6 ± 3.9	< 0.0001
Baseline LH (IU/L)		5.3 ± 2.4	5.4 ± 2.2	0.78
Baseline AMH (ng/mL)		3.2 ± 1.8	0.9 ± 0.4	< 0.0001
AFC		22.5 ± 8.1	9.7 ± 3.5	< 0.0001
LH suppression	Agonist	4 (5)	4 (5)	1
	Antagonist	78 (95)	82 (95)	
Total dose of FSH administered (UI)		1946 ± 603.7	3238.9 ± 1152.2	< 0.0001
Number of OCCs retrieved		12.5 ± 6.7	4.6 ± 3.1	< 0.0001

*Note:* The non‐parametric Mann–Whitney test was used for quantitative variables and the Fisher's Exact Test for qualitative variables with GraphPad Prism, v.8.0 (GraphPad Software, San Diego, CA, USA). Differences were considered significant at *p‐*value < 0.05. Data are expressed as *n* (%), percentage or mean ± SD.

Abbreviations: AFC, antral follicle count; AMH, anti‐Müllerian hormone; BMI, body mass index; FSH, follicle‐stimulating hormone; LH, luteinizing hormone; OA, ovarian ageing profile; OCC, oocyte cumulus complex; SD, standard deviation.

### 3.2 Data Analysis

**FIGURE 2:**
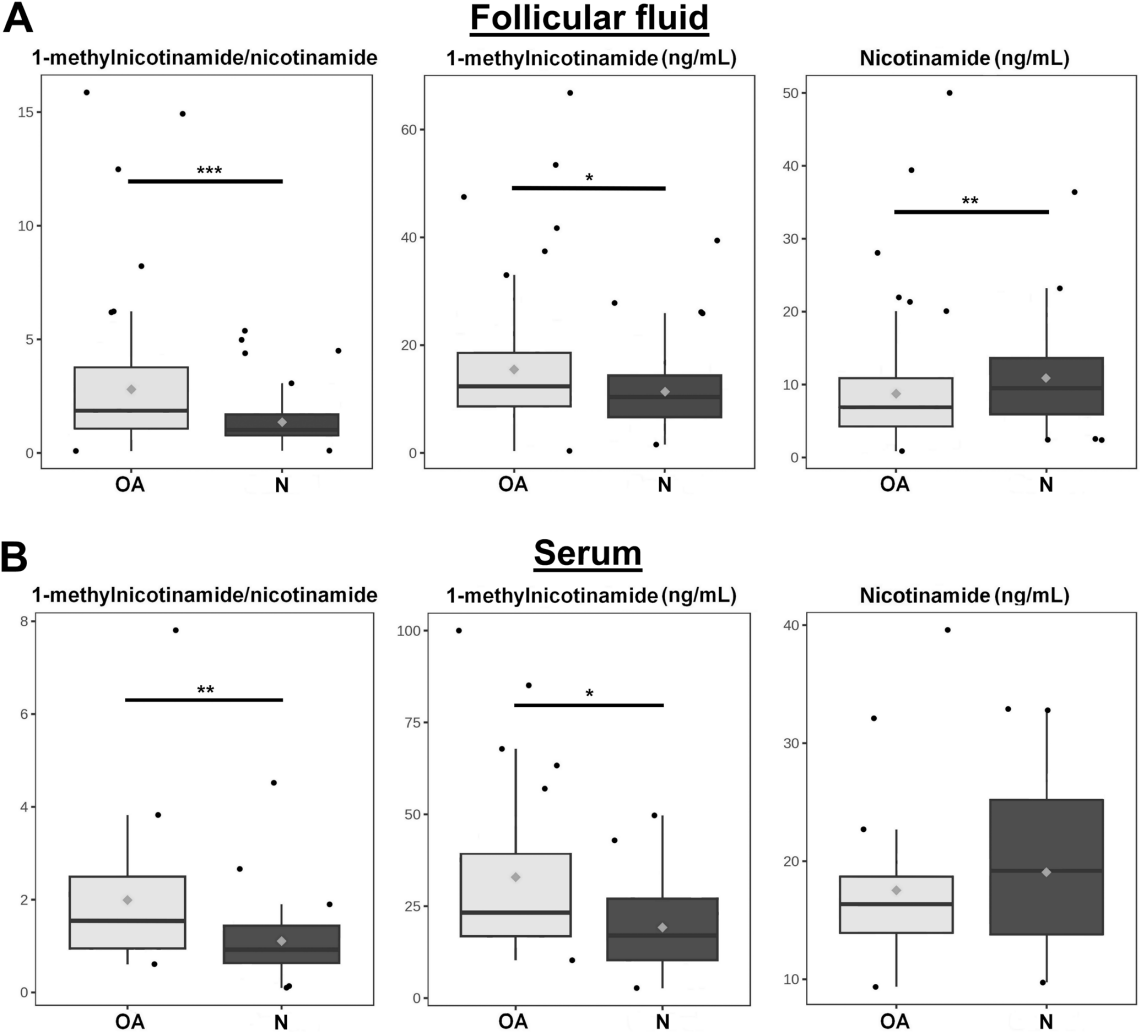
Follicular fluid and serum, raw data. (A) Distributions of concentration between the two follicular fluid cohorts (light grey: Ovarian ageing; dark grey: Controls) are illustrated with significant differences. (B) Distributions of concentration between the two serum cohorts (light grey: Ovarian ageing; dark grey: Controls) are illustrated with significant differences (Wilcoxon rank‐sum test: ***p‐value < 0.001; **p‐value < 0.01; *p‐value < 0.05).

Analysis was conducted on the cohort of 168 FF samples (82 controls and 86 patients with OA) and 53 serums samples (29 controls and 24 patients with OA), leading to the results summarised in Table 3 and illustrated in Figure 2. In FF of OA patients, nicotinamide was found to be significantly decrease (−19%, *p* = 0.002), whereas the 1‐methylnicotinamide concentration was significantly increased (+36%, *p* = 0.014), with a + 100% increased resulting MNA/NAM ratio (*p* < 0.001). The ratio (+80%, *p* = 0.003) and 1‐methylnicotinamide (+71%, *p* = 0.02) were also significantly increased in the serum of OA patients compared to controls, but nicotinamide was no longer significantly altered in blood (*p* = 0.421).

**TABLE 3:** Follicular fluids and serum results: Raw data.

Matrix	1‐methylnicotinamide/nicotinamide		1‐methylnicotinamide (ng/mL)		Nicotinamide (ng/mL)	
	FC	*p*	FC	*p*	FC	*p*
Follicular fluid (*n* = 168)	2.0663	2.40E‐05	1.3601	0.014424	0.80737	0.0021632
Serum (*n* = 53)	1.8004	0.0040139	1.7117	0.020176	0.91831	0.42126

*Note:* For nicotinamide, its methylated form and the ratio, the fold change (FC, ovarian ageing vs. controls) and *p‐*values (Wilcoxon rank‐sum test) are indicated for both matrices.

**FIGURE 3:**
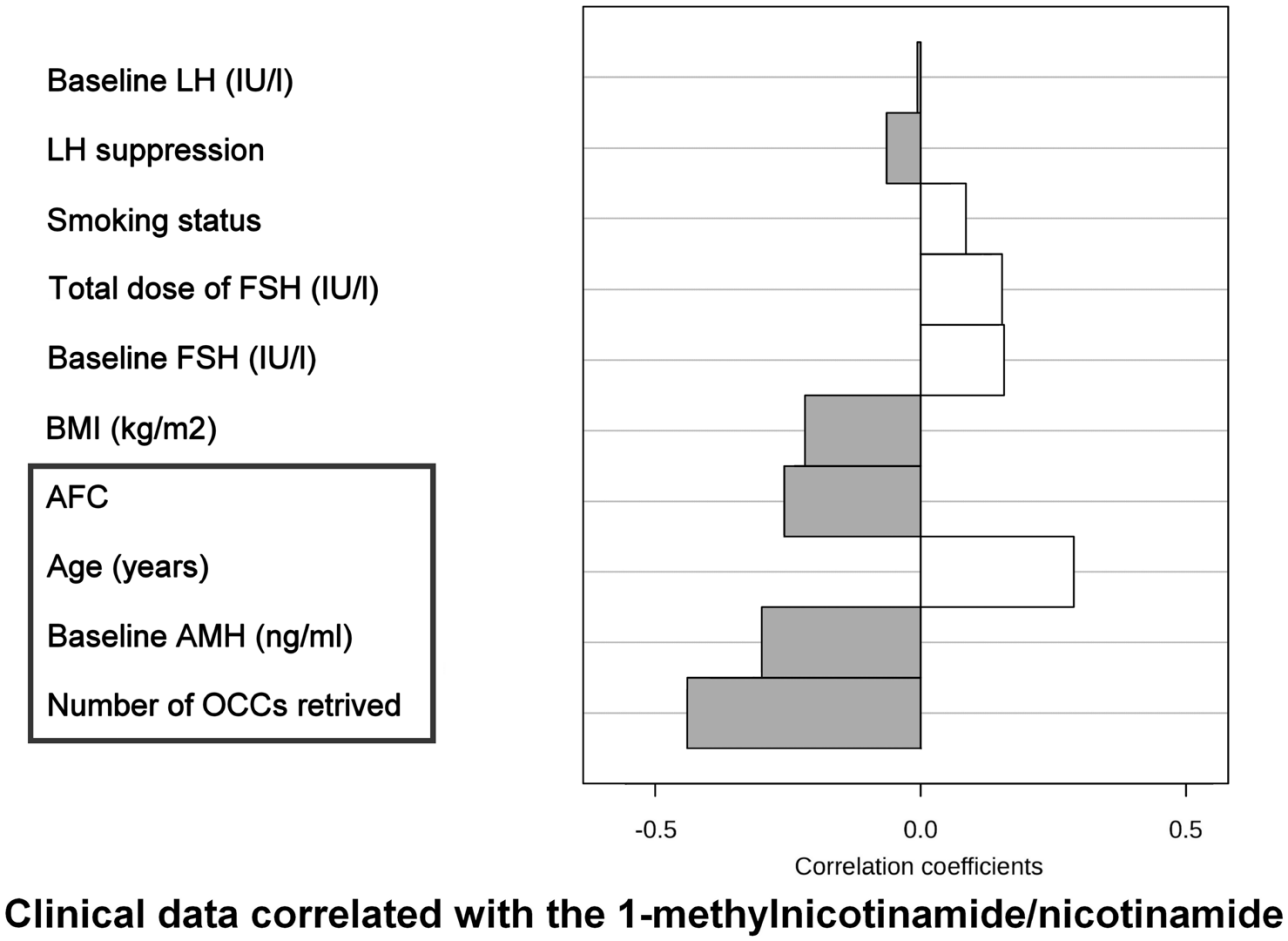
Correlation analysis between clinical data and raw results. Correlations between the parameters quantified in follicular fluids and clinical data are showed, dark grey histograms indicating a negative correlation, white histograms a positive one. Strongest correlations (correlation coefficient > 0.3) are emphasised with a black square.

A correlation study between quantified metabolites in FF and clinical data was conducted (Figure 3). Several factors related to the OA were found to be associated with nicotinamide and, even more, with the MNA/NAM ratio. In fact, with the latter, the number of OCCs retrieved, AMH and AFC appear to be negatively correlated while age showed a positive correlation. Conversely, no significant correlation was identified between the ratio and baseline levels of LH and FSH, body mass index (BMI), tobacco use, or stimulation parameters, including the type of suppression protocol and the administered FSH dose.

A similar analysis was performed between quantified metabolites in serum and clinical data. As with FF, several factors related to ovarian ageing were also found to be associated with nicotinamide and the MNA/NAM ratio. In fact, in serum, the number of OCCs retrieved, AMH and AFC appear to be negatively correlated while age showed a positive correlation.

### 3.3 Correlation Between FF and Blood

A positive correlation has been confirmed between the MNA/NAM ratio in serum and the FF one (corr coef of 0.64 and *p* < 0.001).

### 3.4 The Age Effect

To assess the potential confounding effect of age on the relationship between ovarian function and the 1‐methylnicotinamide/nicotinamide (MNA/NAM) ratio, we performed a series of statistical analyses, including linearity regression and collinearity diagnostics, and propensity score matching.

First, we built a linear regression model that included both variables. The Variance Inflation Factor (VIF) for age and ovarian function was 1.353 (below the threshold of 5), indicating no multicollinearity (Table S1). In this model, ovarian status emerged as a significant predictor of the MNA/NAM ratio (*p* = 0.0005), whereas age was not significantly associated with the MNA/NAM ratio (*p* = 0.7740). These findings suggest that the MNA/NAM ratio is primarily driven by ovarian function, than age.

To ensure robustness, we employed propensity score matching with a calliper of 0.2 to balance age distributions between the OA and N groups. After matching and selecting 49 patients per group, no significant difference in age was observed (*p* = 0.5511, Mann–Whitney test; Table S2). In the age‐matched sample, the MNA/NAM ratio remained significantly higher in the OA group compared to the N group (*p* = 0.0085, Mann–Whitney test), with the same fold change (FC = 2.06 post‐matching vs. FC = 2.07 pre‐matching). This persistent difference after age matching provides strong evidence that the observed association between ovarian function and the MNA/NAM ratio is independent of age.

## 4 Discussion

To our knowledge, this is the first reported quantification of nicotinamide and 1‐methylnicotinamide levels in follicular fluid (FF) specifically in the context of ovarian ageing. We observed a significant reduction in nicotinamide concentration in the FF of patients with ovarian ageing, alongside an increase in 1‐methylnicotinamide levels, resulting in a marked elevation of the MNA/NAM ratio in these patients. These findings are consistent with previous research on oocyte quality. In 2021, Song et al. conducted a non‐targeted metabolomic analysis of serum from poor ovarian responders undergoing IVF and highlighted the involvement of nicotinamide metabolism in this condition [13]. In 2022, Guo quantified nicotinamide in the FF of 46 patients undergoing IVF and reported a positive correlation between its concentration, follicular size, and oocyte quality (maturity and fertilisation potential) [12].

The oxidised and reduced forms, NAD^+^/NADH, function as ubiquitous redox cofactors regulating dehydrogenases involved in key energy‐producing pathways, including glycolysis, the Krebs cycle and β‐oxidation. Beyond its role in energy metabolism, NAD^+^ also acts as a cofactor for several enzymes such as sirtuins, poly(ADP‐ribose) polymerases (PARPs), ADP‐ribosyltransferases (ARTs), CD38 and RNA polymerases [23], all of which are essential for maintaining cellular homeostasis. Through these interactions, NAD^+^ contributes to mitochondrial biogenesis, DNA repair, epigenetic regulation and post‐translational modifications [24, 25]. The age‐related decline in NAD levels across various tissues is well‐documented in both animal models and humans [5, 26]. The role of NAD metabolism in ovarian ageing has also been recognised [27]. The decline in NAD in this context is associated with mitochondrial dysfunction and increased oxidative stress [3, 28], which compromise the activity of NAD‐dependent enzymes involved in genome maintenance and repair [29, 30], as well as in epigenetic regulation. These alterations contribute to meiotic spindle abnormalities [31] and epigenetic modifications [32, 33, 34] that affect oocyte quality during ageing. In murine models, reduced ovarian NAD levels have been directly linked to impaired oocyte competence in the context of ovarian ageing [7, 8]. More recently, deletion of enzymes involved in de novo NAD biosynthesis has been shown to accelerate ovarian ageing [9]. Nevertheless, direct and accurate quantification of NAD metabolism in human oocytes or follicular fluid remains technically challenging. NAD is released extracellularly in very low amounts and is rapidly catabolised into biologically active derivatives such as nicotinamide and 1‐methylnicotinamide [10].

**FIGURE 4:**
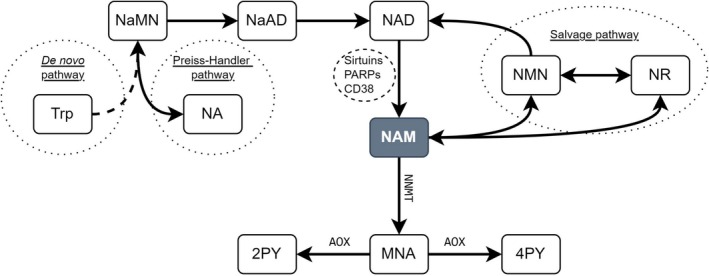
Nicotinamide pathway. Schema summarising key metabolites, enzymes, and pathways nearby nicotinamide. 2PY, N1‐methyl‐4‐pyridone‐5‐carboxamide; 4PY, N‐methyl‐4‐pyridone‐5‐carboxamide; MNA, 1‐methylnicotinamide; NA, nicotinate; NaAD, nicotinate adenine dinucleotide; NAD, nicotinamide adenine dinucleotide; NAM, nicotinamide; NaMN, nicotinate mononucleotide; NMN, nicotinamide mononucleotide; NR, nicotinamide riboside; Trp, tryptophan.

Physiologically, NAD is derived either from *de novo* synthesis from tryptophan, the Preiss‐Handler pathway (nicotinate), or the salvage pathway from nicotinamide derivatives (Figure 4). The salvage pathway is essential for maintaining the NAD pool, which is largely consumed by enzymes such as PARP and CD38 [35].

Numerous studies have proposed the use of NAD^+^ precursors to counteract NAD^+^ deficiency and improve fertility during ageing. Oral administration of nicotinamide mononucleotide (NMN) in aged mice has been shown to enhance follicular development [36], restore oocyte NAD^+^ levels and quality, and increase both the number of ovulated oocytes and litter size [7]. Additionally, supplementation of NMN in embryo culture media has been reported to improve embryonic development in mice [7]. Similarly, Miao et al. demonstrated that in vivo NMN supplementation in aged mice improves oocyte quality by restoring mitochondrial function and enhancing meiotic competence, thereby increasing fertilisation rates and embryonic developmental potential [37]. NAD^+^ precursor, nicotinamide riboside (NR), has also been used in aged mice, resulting in elevated intra‐ovarian NAD^+^ levels. This increase is associated with improved follicular growth and higher live birth rates, likely due to enhanced mitochondrial membrane potential, reduced reactive oxygen species (ROS) and fewer meiotic spindle abnormalities [8]. Furthermore, a recent study in rats showed that supplementation with NMN or NR can reverse ovarian ageing by improving mitochondrial fusion‐fission dynamics [38]. In vitro, the addition of nicotinamide during mouse oocyte maturation reduces oxidative stress and enhances oocyte fertilisation capacity and developmental competence [12]. In cattle, low‐dose nicotinamide supplementation during in vitro oocyte maturation similarly improves oocyte competence by reducing oxidative stress and apoptosis, and by enhancing mitochondrial function [39]. Collectively, these findings support the therapeutic potential of NAD^+^ precursors in restoring oocyte competence during ageing [27, 40]. The feasibility of supplementing these molecules in humans has already been demonstrated in the context of other pathologies [41, 42], and several clinical trials are currently underway [27]. The decrease in nicotinamide levels in follicular fluid observed in OA patients in our study may support its potential use to counteract the effects of ovarian ageing, but will require preliminary pharmacological studies to confirm this hypothesis.

The decrease in NAM could result from insufficient intake, impaired regeneration or overconsumption. In parallel with the decrease in NAM, our study shows an increase in 1‐methylnicotinamide (MNA) in the FF of OA patients. This compound is derived from the degradation of NAM through the action of Nicotinamide N‐methyl transferase (NNMT). It has been described as potentially initiating a stress resistance pathway and exerting a protective effect against ovarian ageing via activation of 5′‐Adenosine Monophosphate‐activated Protein Kinase (AMPK) after oxidation by the aldehyde oxidase 1 (AOX1) [43]. The increase in the MNA/NAM ratio in the FF of these patients would suggest an imbalance favouring the NAM degradation pathway, potentially to the detriment of the NAD salvage pathway, the decline of which is associated with ageing.

The correlations study between the MNA/NAM ratio in the FF shows a negative correlation with AFC, AMH level and the number of oocytes retrieved, while age showed a positive correlation. Additionally, there is a positive correlation between the ratio values in FF and serum. In the latter matrix, the same correlations as in the FF are found between the MNA/NAM ratio and clinical parameters, that is, AMH, AFC and number of oocytes retrieved. These parameters are all markers of OA. It would be worthwhile to prospectively study the serum MNA/NAM ratio as a biomarker of OA and as a predictor of the number of oocytes obtained in IVF. Larger studies should also investigate its influence on the outcome of IVF attempts. Interestingly, the MNA/NAM ratio seems primarily driven by OA profile rather than just chronological age, reinforcing its relevance to reflect OA independently of chronological age.

Our study shows an association between altered nicotinamide metabolism and ovarian ageing. Although further studies are needed to establish a causal relationship, this association may be related to mitochondrial dysfunction, which has been implicated in the decline of oocyte quality observed during ovarian ageing. In the future, it would be of interest to evaluate the potential of nicotinamide both as a biomarker and as a therapeutic agent for mitochondrial dysfunction in this clinical context.

## Author Contributions

**Cinzia Bocca:** conceptualization (equal), data curation (equal), formal analysis (equal), investigation (equal), methodology (equal), validation (equal), writing – original draft (equal). **Magalie Boguenet:** conceptualization (equal), data curation (equal), formal analysis (equal), investigation (equal), methodology (equal), writing – original draft (equal), writing – review and editing (equal). **Lisa Boucret:** data curation (equal), methodology (equal), writing – review and editing (equal). **Pierre‐Emmanuel Bouet:** data curation (equal), methodology (equal), writing – review and editing (equal). **Pascal Reynier:** conceptualization (equal), data curation (equal), formal analysis (equal), funding acquisition (equal), investigation (equal), methodology (equal), project administration (equal), writing – original draft (equal). **Pascale May‐Panloup:** conceptualization (equal), data curation (equal), formal analysis (equal), funding acquisition (equal), investigation (equal), methodology (equal), project administration (equal), validation (equal), writing – original draft (equal).

## Funding

The authors have nothing to report.

## Conflicts of Interest

The authors declare no conflicts of interest.

## Supporting information


**Table S1:** Linear regression results for the 1‐Methylnicotinamide/Nicotinamide (MNA/NAM) ratio.
**Table S2:** Balance assessment of age before and after propensity score matching (Calliper = 0.2).

## Data Availability

Data will be made available upon request to the corresponding author at this address pamaypanloup@chu-angers.fr.
