# Supplementary material for: Nicotinamide (Vitamin B3) Deficiency in Follicular Fluid of Patients With Ovarian Ageing
Source: J Cell Mol Med. 2026 Mar 19;30(6):e71085. doi: 10.1111/jcmm.71085 (PMC13098092; doi:10.1111/jcmm.71085)
Supplement: Supplementary file 1 — Table S1: Linear regression results for the 1‐Methylnicotinamide/Nicotinamide (MNA/NAM) ratio. Table S2: Balance assessment of age before and after propensity score matching (Calliper = 0.2). [file JCMM-30-e71085-s001.docx]

**Supplementary table 1. Linear regression results for the 1-Methylnicotinamide/Nicotinamide (MNA/NAM) ratio.** VIF = Variance Inflation Factor. SE = Standard Error. CI = Confidence intervals. Condition: Ovarian status (OA: ovarian aging; N: normal). DF = Degrees of Freedom.

| Variable | Estimate | SE | 95% CI | *p*-value | VIF | R² | 0.1006 |
| --- | --- | --- | --- | --- | --- | --- | --- |
| Intercept | 0.9427 | 1.453 | -1.926 to 3.811 | 0.5174 | - | **DF** | 165 |
| Condition (OA - N) | 1.388 | 0.3924 | 0.6133 to 2.163 | 0.0005 | 1.353 | **F (DFn, DFd)** | F(2, 165) = 9.223 |
| Age (years) | 0.01269 | 0.04410 | -0.0744 to 0.09977 | 0.7740 | 1.353 | ***p*-value (ANOVA)** | 0.0002 |

**Supplementary table 2. Balance assessment of age before and after propensity score matching (Caliper = 0.2). SMD = Standardized Mean Difference. eCDF = Empirical Cumulative Distribution Function.** OA = ovarian aging. N = normal. The non-parametric Mann-Whitney test was used with GraphPad Prism, v.8.0 (GraphPad Software, San Diego, CA, USA). Differences were considered significant at *p*-value < 0.05. Age, in the two groups, before and after matching, is expressed as mean ± standard deviation (SD). The fold change (FC, ovarian ageing *vs.* controls) of the 1-methylnicotinamide/nicotinamide (MNA/NAM ratio), before and after matching is also provided.

| Metric | Before Matching | After Matching |
| --- | --- | --- |
| Standardized Mean Difference (Age) | -1.2025 | -0.1285 |
| eCDF Mean (Age) | 0.2999 | 0.0314 |
| eCDF Max (Age) | 0.4932 | 0.1429 |
| Sample sizes (N/OA) | 82/86 | 49/49 |
| Mean age (N/OA) | 32.3±3.8/36.6±4 | 34.07±3.5/34.56±3.2 |
| *p*-value (Age) | < 0.0001 | 0.5511 |
| FC (MNA/NAM ratio) | 2.07 | 2.06 |
| *p*-value (MNA/NAM ratio) | < 0.0001 | 0.0085 |
